# Development and validation of the Dog Obesity Risk and Appetite (DORA) questionnaire

**DOI:** 10.1186/1751-0147-57-S1-O18

**Published:** 2015-09-25

**Authors:** Eleanor Raffan, Sarah Diss, Stephen Smith, Jane Wardle

**Affiliations:** 1 Institute of Metabolic Science, University of Cambridge, Cambridge, UK; 2 Department of Clinical Pharmacology, Addenbrooke's Hospital, Cambridge, UK; 3 Institute of Epidemiology & Health, University College London, London, UK

## Introduction

Owner mismanagement is often cited as the cause of canine obesity but it is possible some dogs have higher drives to seek out and eat food than others.

## Objectives

To develop an owner-reported measure of canine appetitive behaviour, and owner- or dog-related factors that influence the development of obesity.

## Methods

Owner interviews, literature review, and questionnaires on similar topics for children were used to identify themes and generate items. Following a pilot phase, a 75 item questionnaire was administered to 302 owners. Factor structure and descriptive statistics were generated and results compared with semi-structured interview responses with a subset of respondents. Optimum questions contributed to the final 34 item questionnaire taken by 261 owners, repeated by 79 two weeks later to assess test-retest reliability. Associations with owner-assigned body condition score were tested.

## Results

The final questionnaire was reliable, and had a clear factor structure, with 3 dog factors (food responsiveness and satiety, lack of selectivity, interest in food), 4 owner factors (owner motivation, owner intervention, restriction of human food, exercise taken), and two dog health factors (signs of gastrointestinal disease, current disease). Results showed that high scores on dog factors and low scores on owner factors were associated with significantly higher condition scores, validating the results of the questionnaire.

## Conclusions

The DORA questionnaire is a reliable, informative owner-reported measure of canine eating behaviour, owner management, and dog health factors that might affect the development of obesity, applicable to studying canine obesity and to clinical veterinarians.

